# The Vocal Extent Measure: Development of a Novel Parameter in Voice Diagnostics and Initial Clinical Experience

**DOI:** 10.1155/2018/3836714

**Published:** 2018-03-04

**Authors:** Philipp P. Caffier, Andreas Möller, Eleanor Forbes, Constanze Müller, Marie-Louise Freymann, Tadeus Nawka

**Affiliations:** ^1^Department of Audiology and Phoniatrics, Charité-University Medicine Berlin, Campus Charité Mitte, Charitéplatz 1, 10117 Berlin, Germany; ^2^Max-Planck Institute for Plasma Physics, Wendelsteinstraße 1, 17491 Greifswald, Germany; ^3^University of Music Carl Maria von Weber Dresden, Wettiner Platz 13, 01067 Dresden, Germany

## Abstract

Voice range profile (VRP) and evaluation using the dysphonia severity index (DSI) represent essentials of instrument-based objective voice diagnostics and are implemented in different standardized registration programs. The respective measurement results, however, show differences. The aim of the study was to prove these differences statistically and to develop a new parameter, the Vocal Extent Measure (VEM), which is not influenced by the measurement program. VRPs of 97 subjects were recorded by two examiners using the established registration programs DiVAS (XION medical) and LingWAVES (WEVOSYS) simultaneously. The VEM was developed on the basis of VRP area and perimeter. All 194 VRP files were analyzed for various parameters and gender independence. The registration programs exhibited significant differences in several vocal parameters. A significant gender influence for DSI was found with DiVAS (*p* < 0.01), but not with LingWAVES. The VEM quantified the dynamic performance and frequency range by a unidimensional, interval-scaled value without unit, mostly between 0 and 120. This novel parameter represents an intelligible and user-friendly positive measure of vocal function, allows simple and stable VRP description, and seems to be suitable for quantification of vocal capacity. In contrast to DSI, the VEM proved to be less susceptible to registration program and gender.

## 1. Introduction

A comprehensive evaluation of the quality and capability of the human speaking and singing voice is difficult due to its multidimensional nature and several influencing factors including subjective esthetic aspects and distracting semantic or nonsemantic information [1–3]. Sound production within the voice box is a highly complex phenomenon whose oscillating and muscular mechanisms can be investigated and visualized at the glottal level [4–6]. However, various other regions of the human body and further essential elements are known to have a relevant impact on vocal performance, amongst others: (1) breathing-related issues for generation of the airflow and sufficient subglottal pressure for vocal fold vibrations [7–9], (2) training-induced optimized utilization of physiologically and anatomically given conditions for resonance processes, sound amplification, and refinement in the vocal tract [10–12], (3) peripheral and central hearing function, perception, and neurological mechanisms of vocal control including auditory and kinesthetic feedback [13–17], (4) presence of psychologically influencing factors, stressful conditions, and current mental and emotional state [18–20], and (5) physical fitness and constitution including effects of body tension, posture, movements, and physical load during phonation [21–24]. Resulting from all that, since voice quality is not clearly defined and a multidimensional perceived construct, several measurements are recommended for the current state-of-the-art evaluation of vocal function [2, 25]. These measurements comprise subjective procedures such as auditory-perceptual judgment [26] and patients' self-assessment of voice [27], as well as objective procedures such as frequency-dependent measurements of the sound pressure level (voice range profile [VRP] measurement), acoustic-aerodynamic analysis, and videolaryngostroboscopy.

In objective voice diagnostics, specific algorithms are applied to quantify certain aspects of a correlate of voice production. By measuring a series of spoken words and singing tones at the softest and the loudest phonation possible, the resulting speaking-voice and singing-voice profiles yield a quick and clear mapping of the vocal capacity (“audiogram” of the voice). The standardized VRP and the data thereby ascertained in order to calculate the established dysphonia severity index (DSI) are core elements in instrument-based phoniatric assessment [28, 29]. Computer-assisted application of the lowest intensity, the highest tone, the maximum phonation time, and jitter is implemented in different standardized registration programs such as DiVAS (XION medical, Berlin, Germany) and LingWAVES (WEVOSYS, Forchheim, Germany). Though both programs represent modern, reliable, and internationally acknowledged systems for speech and voice assessment, the measurement results of both programs reveal differences in clinical practice. Therefore, one objective of this study was to clarify whether these differences could be verified statistically. In addition, our aim was to develop a new parameter which is less influenced by the registration programs. Since the established DSI quantifies dysphonia as a negative criterion and involves the risk of inaccurate results due to its multidimensional acquisition, our intention was to create and present a one-dimensional, intelligible, and user-friendly positive measure of vocal range. This novel parameter, the Vocal Extent Measure (VEM), was designed for objective VRP evaluation and quantification of vocal performance.

## 2. Materials and Methods

**Figure 1:**
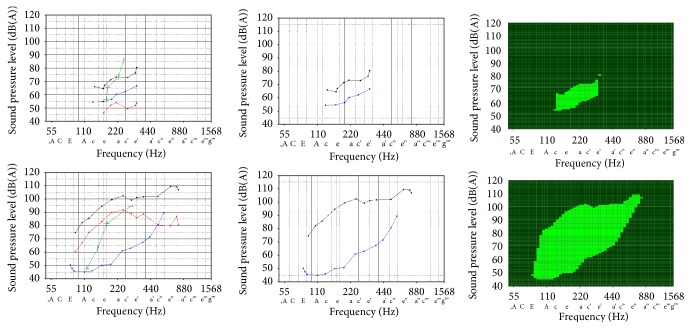
Results of voice range profile (VRP) measurements with the DiVAS program. The upper row shows a small VRP in one patient with reduced vocal capacity due to edema of both vocal folds. The lower row displays a large VRP in a healthy singer with high vocal capacity. Left: VRPs with graphical presentation of all relevant parameters: SPL of speaking voice (green curve) at different vocal intensity levels, SPL of softest singing (blue curve), SPL of loudest singing (black curve), and singer's formant level (red curve, characterizing the concentration of acoustic energy by resonator amplification of certain frequency ranges in the vocal tract). Middle: extraction of the envelope curves, showing the maximum limits of the softest singing voice (blue, lower line) and the loudest singing voice (black, upper line). Right: closed polygon representation of the VRP area after linear interpolation of missing measurement values as the basis for the VEM calculation (AVA program).

All VRPs were recorded in the sound-treated voice lab of our department with a background noise < 40 dB(A) in accordance with the recommendations of the European Laryngological Society [25, 30]. The measured values were documented in a coordinate system (see Figure 1, left) where the abscissa displays the fundamental frequency in Hz and the musical pitch notation (36 mm for one octave) and the ordinate shows the sound pressure level (SPL; 15 mm for 10 dB). The abscissa is scaled to semitones to ensure a comparable evaluation of male, female, and children's voices with different VRPs. The measurements started with the assessment of speaking-voice profiles (green crosses or curve-shaped presentation). Participants had to speak number series (from 21 upwards, in German) at different vocal intensity levels. After unstressed phonation at indifferent pitch using the lowest volume possible, the schedule of increments comprised the normal “narrator voice” (fist level of increase) and the “lecture voice” (second level of increase) up to the loudest speaking volume (“calling voice”). Subsequently, singing-voice profiles were recorded over the whole vocal pitch range, each as softly (blue dots/curve) and after that as loud as possible (black dots/curve). For both settings, participants began at their middle pitch and went down to the lower limit of their vocal range and thereafter up to the high-level peak pitches. For the loudest-sung tones, the SPL was simultaneously recorded in the spectral range of 2–4 kHz, displaying the special energy components of the singer's formant level (red dots/curve).

The new objective VEM parameter was to be calculated on the basis of the area and the perimeter of the VRP, displayed by the curves for the softest and the loudest singing (Figure 1, middle). Missing values between the lowest and highest tones of the soft and loud singing curve were linearly interpolated, resulting in a closed polygon (Figure 1, right). The fundamental idea was that an ideal VRP shape should not show abrupt differences in the dynamic range of notes produced by the patients along their frequency range. A well-balanced dynamic extent approximates the shape of VRP to a circle, in which, compared to other geometric figures, the area is biggest for a given perimeter. The dynamic range is evenly distributed over the tonal extent in this ideal conception. Each deviation from the circular shape indicates a decrease of vocal performance. Hence, the VEM multiplies the VRP area by the quotient of the measured perimeter of the profile and the theoretical perimeter of a circle with the same area as the profile itself. The construction and mathematical derivation of the VEM measure comprises the following equations and calculations.

The area (*A*) of a circle is defined as

(1) $$\begin{array}{c} A_{circle}=\pi r^{2}. \end{array}$$

The perimeter (*P*) of a circle is defined as

(2) $$\begin{array}{c} P_{circle}=2\pi r. \end{array}$$

Thus, the perimeter of a circle with a known area is described as

(3) $$\begin{array}{c} P_{circle}=2\pi \sqrt{\frac{A_{circle}}{\pi }}. \end{array}$$

The VEM is the product of the VRP area and the quotient of the actual VRP circumference and the perimeter of a circle with the VRP surface area:

(4) $$\begin{array}{c} {VEM}_{exp}=A_{VRP}\frac{P_{circle}}{P_{VRP}}=A_{VRP}\frac{2\pi \sqrt{A_{circle}/\pi }}{P_{VRP}}. \end{array}$$

However, the resulting exponential characteristic is unfavorable for correlations with linear variables. Simple logarithmical conversion yields unpractical small values. Therefore, a coefficient and a subtrahend have been added finally:

(5) $$\begin{array}{c} VEM=50\ln\left(\;A_{VRP}\frac{2\pi \sqrt{A_{circle}/\pi }}{P_{VRP}}\;\right)-200. \end{array}$$

Calculation of the VEM was done after VRP measurement by a proprietary software program (AVA) which can extract various other parameters from the VRP, thereby enabling VRP comparisons [31].

After development of the VEM measure, its initial clinical application was investigated by VRP comparison registered with two different programs for objective voice diagnostics. The VRPs of 97 subjects were recorded simultaneously by two experienced examiners under practically identical conditions. One examiner used the DiVAS program (XION medical, Berlin, Germany) and the other the LingWAVES program (WEVOSYS, Forchheim, Germany). The external microphones of both systems measured the speaking and singing voice in real time under defined and reproducible conditions. The XION headset guaranteed a fixed position of the electret microphone with a distance from the lips of 30 cm. An integrated electronic circuitry calibrated the microphone connection automatically. A USB plug connected the microphone headset to the computer without requiring further adjustments for the acoustic recordings. The WEVOSYS hardware (IEC 651 Type 2, ANSI S1.4 Type 2) was placed at the same distance from the lips. The SPL meter used in this system represents a high-quality microphone, which was connected to the computer through a free USB port and automatically configured by the software.

All patients were consecutively presenting at the outpatient clinic of our phoniatric department. The measurement of their vocal capacity was part of a routine appointment. Besides the VRP measurements and VEM calculations, the voices of all participants were categorized according to the RBH system, where the perceived patient's roughness (R), breathiness (B), and overall hoarseness (H) have to be scored on a scale from 0 to 3 (0 = not existing, 1 = mild, 2 = moderate, and 3 = severe). The RBH-status was detected on the basis of the standardized text “the north wind and the sun” (German version). In addition, digital videolaryngostroboscopy was carried out for classification of laryngeal findings [32] using a high-resolution rigid videolaryngoscope with integrated microphone connected to the Endo-STROB control unit (XION medical, Berlin, Germany).

All resulting 194 VRP files were put through standardized evaluation by a software program which registered the new parameter VEM as well as the established DSI, the maximum phonation time (MPT), jitter (in %), the highest tone (*F*0_high_), the lowest tone (*F*0_low_), frequency range (*F*0_max_), the lowest intensity (*I*_low_), the highest intensity (*I*_high_), dynamic range (*I*_max_), mean dynamic range per semitone (*I*_mean_), area of the VRP (*A*_VRP_), and perimeter of VRP (*P*_VRP_). These parameters were compared with each other, correlated with the subjective hoarseness (H) assessment according to the RBH scale, and examined for gender-specific differences. Statistical methods used were the single factor analysis of variance (ANOVA), the *t*-test for independent and paired samples, and the calculation of Pearson's and Spearman's correlation coefficients (*r*) for interval or ordinal scaled values.

## 3. Results

The study cohort (*n* = 97) included 65 female and 32 male subjects. Their age ranged between 12 and 75 years (44 ± 17 years [mean ± SD]). Videolaryngostroboscopy revealed in 61 patients various organic diseases at vocal fold level. Classification of the resulting organic dysphonia according to the underlying pathology revealed in 24 patients (39%) diseases of the lamina propria (e.g., nodules, polyps, cysts, and oedema), in 21 patients (35%) movement disorders (e.g., vocal fold paralysis), in 10 patients (16%) diseases of the epithelium (e.g., leukoplakia, hyperkeratosis, carcinoma (in situ), and papillomatosis), and in 6 patients (10%) arytenoid pathologies (e.g., granuloma). Thirty-six participants had a normal laryngeal anatomy, 29 of whom suffered from a vocal load induced functional dysphonia, while 7 subjects had normal voices without any complaints. Altogether, 38 subjects had no hoarseness (H0), including 7 healthy participants, 29 patients with functional dysphonia, and 2 patients with organic findings distant from the vocal fold level (small arytenoid granuloma). Forty-three individuals used their voice in a nonprofessional manner (e.g., business (wo)men, clerks, and laborers), whereas 54 patients had a high vocal strain in their profession (e.g., teachers, lecturers, actors, and singers). Subjects of both sexes were comparable in terms of age, level of hoarseness (H), underlying pathologies, and sociodemographic characteristics (see Table 1 for details).

As planned, the VRP measurements were successfully executed in all study participants with both registration programs simultaneously, altogether resulting in 194 VRPs (97 DiVAS and 97 LingWAVES). Hence, for each subject, there were 2 VRPs available for further analysis. The AVA software realized calculation of the new parameter VEM in all cases without any additional effort or loss of time. The VEM quantified the patient's dynamic performance and frequency range by a unidimensional, interval-scaled value without unit in the range between about −150 and +150, mostly between 0 and 120. A large VRP with high vocal capacity was characterized by a high VEM value; conversely, a small VRP resulted in a small VEM.

The comparison of the recordings with the LingWAVES and DiVAS program revealed diverging results for the different parameters investigated (Table 2). From all registered parameters, only MPT was exactly identical in both programs. For VEM, DSI, and *F*0_low_, no significant differences were found (*p* > 0.05). However, the values for jitter and *I*_high_ (*p* < 0.05) as well as the frequency range *F*0_max_ (*p* < 0.01) differed significantly. All other parameters examined showed a highly significant difference (*p* < 0.001).

**Figure 2:**
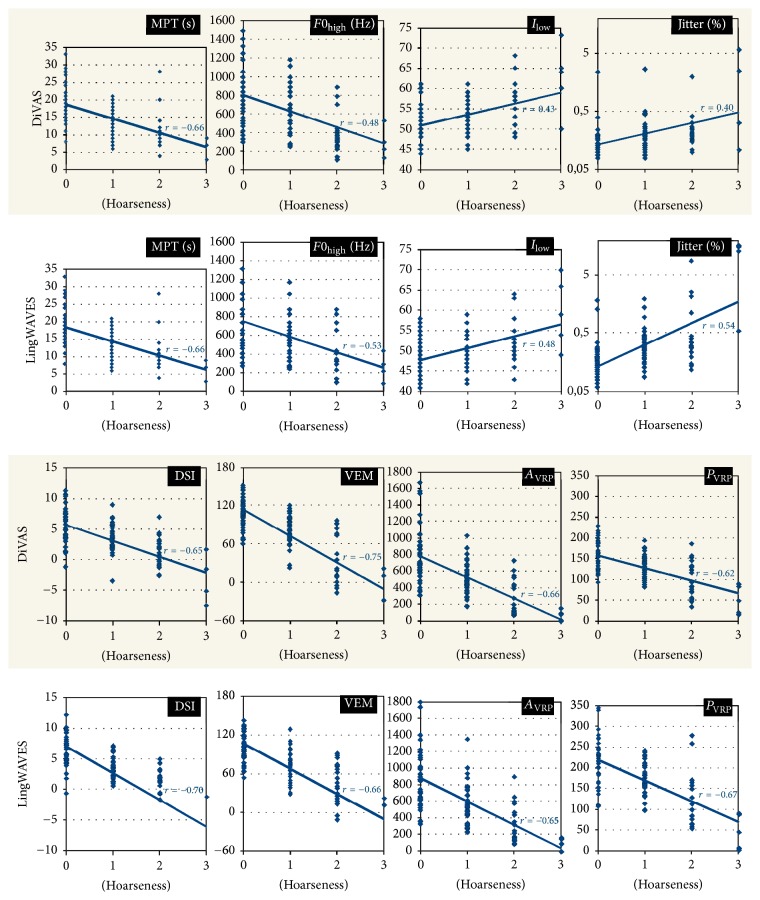
Comparison of the programs DiVAS and LingWAVES for selected parameters (MPT, *F*0_high_, *I*_low_, jitter, DSI, VEM, *A*_VRP_, and *P*_VRP_) and their correlation with the level of hoarseness (H).

The direct program comparison for selected parameters including Spearman's correlations with the degree of hoarseness (H) is graphically displayed in Figure 2. The VEM and H correlated highly significantly (*p* < 0.001) at an average of *r* = −0.71 (DiVAS *r* = −0.75; LingWAVES *r* = −0.66). Likewise, the DSI correlated highly significantly with H at an average of *r* = −0.67 (DiVAS *r* = −0.65; LingWAVES *r* = −0.70). Concerning the total study group, DSI and VEM registration with DiVAS offered higher mean values compared to LingWAVES (3.4 versus 3.2; 77 versus 72). This was also true for the subgroups with the hoarseness levels H1 (2.9 versus 2.5; 72 versus 68), H2 (0.4 versus −1.2; 40 versus 36), and H3 (−2.1 versus −5.8; −12 versus −16). For the H0 patient group without hoarseness, DSI and VEM registration with DiVAS versus LingWAVES showed divergent results (5.8 versus 6.7; 116 versus 112). However, within both registration programs, the mean DSI and VEM values for the hoarseness levels H0, H1, H2, and H3 differed significantly from each other (*p* < 0.001).

**Figure 3:**
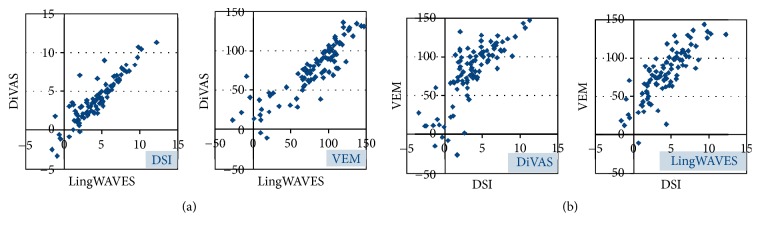
Scatterplots of DSI and VEM. (a) Distribution of both parameters when registered with DiVAS and LingWAVES at the same time. (b) Distribution of values for both parameters within each registration program.

Figure 3 illustrates for both parameters the distribution of values between LingWAVES and DiVAS and within each registration program. Pearson's correlation of VEM between LingWAVES and DiVAS (*r* = 0.87) was approximately equal to the correlation of DSI between both programs (*r* = 0.86). VEM and DSI correlated also with each other highly significantly (*p* < 0.001) at an average of *r* = 0.84 (DiVAS) and *r* = 0.85 (LingWAVES).

**Figure 4:**
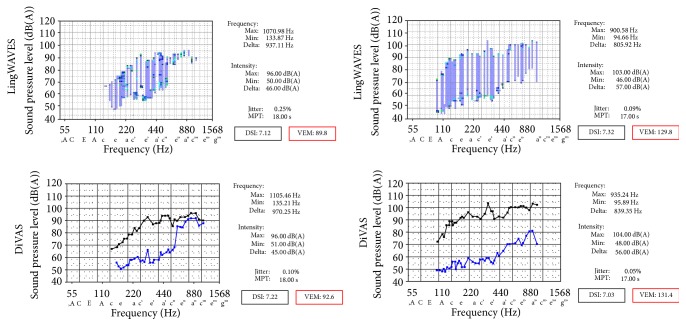
Comparison of VRPs and objective parameters in a female singer (left) and a male singer (right) registered simultaneously with LingWAVES (top) and DiVAS (bottom). The VRPs of both subjects differ considerably and have correspondingly dissimilar values for the VEM (89.9 and 92.6 versus 129.8 and 131.4). In contrast, almost equal DSI scores were obtained (7.12 and 7.22 versus 7.32 and 7.03).

Gender-specific differences proved highly significant (*p* < 0.001) with LingWAVES for the parameters *F*0_low_, *F*0_high_, and frequency range (*F*0_max_). An influence of gender on the parameters VEM, DSI, *I*_low_, *I*_high_, and mean dynamic range per semitone (*I*_mean_) was not detectable (*p* > 0.05). A highly significant gender influence on the parameters *F*0_low_, *F*0_high_, and *F*0_max_ was also found with the DiVAS program (*p* < 0.001). The influence on the DSI proved to be very significant (*p* < 0.01) in contrast to LingWAVES. Gender showed no influence (*p* > 0.05) on the parameters VEM, *I*_low_, *I*_high_, and *I*_mean_. With neither program any significant gender influence on other parameters was found, including MPT (*p* > 0.05). Regarding comparison of vocal performance, the VEM values described the VRP in both genders better than the established DSI.  Figure 4 shows the measurements of a female and a male singer, which were simultaneously recorded with both registration programs. Both subjects had a comparable DSI, but different VRPs and vocal capacities. This example indicates, in contrast to the VEM, the significant influence of the recorded acoustic and aerodynamic parameters on the multidimensional DSI calculation. It illustrates also the differing intention of both parameters, which obviously represent different aspects of vocal function.

## 4. Discussion

In the group of subjects examined, the comparison of the standardized registration programs DiVAS and LingWAVES confirmed the assumed differences of acoustic measurement results in clinical practice. The influence of the software used in computer-assisted phoniatric examination cannot be disregarded, as is clear from the significant differences of simultaneously recorded measurement data from both programs processing identical signals. In spite of these differences, our findings endorse the literature results which prove high reliability and reproducibility of VRP measurements, and thus clinical usefulness [33, 34].

Concerning the established DSI, it is described as a reliable and suitable parameter to measure the severity of dysphonia [35–37]. Our study supports the general opinion that the DSI represents a robust instrument which is useful for dysphonia quantification. It is calculated as a weighted combination of the highest possible frequency (*F*0_high_), the lowest intensity (*I*_low_), MPT, and jitter [29]. Although our data proved that these components, apart from the MPT, are highly dependent on the registration program, the DSI did not show any significant differences when another software was used. Obviously, compensation arises from the multidimensional orientation of the different elements involved in the calculation of this parameter. Nevertheless, it must be noted that the DSI is able to supplement but not to replace the subjective voice assessment, since our data revealed only a moderate negative correlation with the degree of hoarseness (*r* = −0.67). Besides, previous studies indicated that the DSI is influenced not only by differences of measurements due to the registration programs, but also by age and gender [38–40]. Our results confirmed these findings, as in the present group of subjects some gender influence was detected for the DSI with the DiVAS program. To improve the quality of voice diagnostics, we saw the need to develop and investigate the VEM as an objective parameter unimpaired by these confounding factors.

Comprehensive medical voice diagnostics involves various subjective and objective instruments [25, 28, 30]. The pool of objective parameters has the potential of continuous enhancement and development of new devices, due to the advances in measurement technology, modeling, and digital signal processing [41–44]. The aim of this process is to improve, objectify, and standardize the measurements as well as the documentation of vocal function and diagnosis. However, each new parameter has to be investigated critically regarding its new value and necessity. The VEM was developed as a unidimensional score which represents a quantitative correlate of the individual vocal performance. Neither the pure tonal range nor the dynamic range may be suitable to document vocal capability. Therefore, the VEM uses the VRP area as a better correlate for quantification, though the area displays an inaccurate impression of the actual usable voice performance. For example, singers with a large VRP but tight restrictions in their dynamic range on single tones may not be able to use their whole frequency range artistically. For that reason, the VRP perimeter was also incorporated into the calculation of this new parameter. The outcome is a value which registers the subject's dynamic capacity and frequency range, also considering the uniformity of dynamic progression of the VRP limits (i.e., the curves for the softest and loudest singing) as a qualitative vocal characteristic. As described above in detail, the resulting new parameter was constructed as VEM ≈ area (VRP)/rel.perimeter (VRP). Thus, the VEM calculation is not influenced by measurements which are likely to be influenced by age or gender [45, 46]. Elements which are highly susceptible to interference (e.g., jitter) are not considered. The VEM is also independent of the pitch (i.e., women's and men's voices are evaluated in the same way) and the vocal intensity (i.e., microphone distance has no influence on the assessment of the VRP).

According to our results, the VEM provided a useful representation of the recorded VRP and enabled more advanced portability between different systems than VRP data alone. Although a few subjects revealed considerable program-related differences in the VRP recordings, the VEM can be used reliably to document vocal capacity. It is less influenced by differences in measurements and therefore delivers the same results for equivalent vocal performance using different systems. A large total intensity range and a large total fundamental frequency range resulted in a high VEM score. Conversely, a small VRP led to a small VEM. The VEM was scaled in the majority of subjects from 0 to 120; however, it was indeed possible to exceed these boundaries at both ends. Further studies including more severely impaired and exceptionally great voices must show the amount of values in the higher positive and negative range.

Regarding the limitations of our investigation, some characteristics of the study design and methodology have to be taken into account. Firstly, the sample size was too small to ensure a representative distribution of the population, to examine comparably sized hoarseness subgroups, or to be considered representative for diagnosis-related groups of patients to whom the results could be generalized or transferred. Secondly, some general and well-known factors influencing the VRP registration have to be discussed critically, for example, dependence on the experience and routine of the examiner, motivation and musicality of the subject investigated, and the absence of generally accepted specifications regarding the quantity of sung and registered tones. Furthermore, the size of the recorded tone intervals influences the VRP circumference. For example, larger intervals can hide register changes with reduced vocal capability and thus wrongly increase the VRP. Most of these error sources can be neglected in our study, because all VRPs were recorded simultaneously by two equivalently experienced examiners under practically identical conditions.

Our results also demonstrated a different orientation of the VEM compared to the DSI. Whereas the DSI describes the severity of a dysphonia, the VEM represents vocal capacity. Thus, the VEM is also suitable for the documentation of increases in vocal performance in normal and trained voices. This enables a classification of the voice in a positive sense according to performance capacity, instead of emphasizing dysphonia as a negative criterion. In this way, the VEM augments comprehensive voice diagnostics and documentation. Future studies with larger samples of participants are necessary to explore and define intervals relating to the degrees of hoarseness and vocal capacity and thus classify the VEM values.

## 5. Conclusions

The new VEM represents a useful, intelligible, and user-friendly positive measure of vocal function. It is calculated automatically from the VRP and may be easily implemented into existing clinical protocols. This novel parameter quantifies the VRP unidimensionally by a single concrete score, instead of estimating it by means of visual perception and a few exposed values. In contrast to DSI, the VEM proved to be less susceptible to registration program and gender. The VEM allows simple and stable VRP description and seems to be suitable for the quantification of vocal capacity. This new parameter of vocal performance provides additional information about voice function. Therefore, VEM introduction in practical objective voice diagnostics is appropriate and desirable, complementing the established DSI.

## Figures and Tables

**Table 1:** Patient characteristics. Unless otherwise specified, data expressed as number of patients and percentage of group (NA: not applicable).

Characteristics	Number of all patients	% of total group (*n* = 97)	Number of male patients	% of male group (*n* = 32)	Number of female patients	% of female group (*n* = 65)
*Gender*						
Male	32	33%	-	-	-	-
Female	65	67%	-	-	-	-
*Age *						
Years (mean ± SD)	44 ± 17	-	49 ± 16	-	41 ± 17	-
*Sociodemography*						
Scholar	6	6%	1	3%	5	8%
Student/apprentice	11	11%	3	9%	8	12%
Employed	59	61%	21	66%	38	59%
Unemployed	5	5%	1	3%	4	6%
Pensioner	16	17%	6	19%	10	15%
*Main voice use*						
Nonprofessional	43	44%	16	50%	27	42%
Professional	54	56%	16	50%	38	58%
*Dysphonia*						
Functional	29	30%	9	28%	20	31%
Organic	61	63%	22	69%	39	60%
N/A; healthy	7	7%	1	3%	6	9%
*Hoarseness level*						
H0 (not existing)	38	39%	11	34%	27	42%
H1 (mild)	35	36%	8	25%	27	42%
H2 (moderate)	19	20%	10	31%	9	13%
H3 (severe)	5	5%	3	10%	2	3%

**Table 2:** Results of parameters investigated with the 2 registration programs DiVAS (XION medical, Berlin, Germany) and LingWAVES (WEVOSYS, Forchheim, Germany).

Parameter of VRP	LingWAVES (mean ± SD)	DiVAS (mean ± SD)	Significance *p* (*t*-test)	Difference between genders (LingWAVES)	Difference between genders (DiVAS)
VEM	71.73 ± 52.24	76.52 ± 48.4	NS	NS	NS
DSI	3.17 ± 5.55	3.4 ± 3.59	NS	NS	*∗∗*
MPT (s)	14.71 ± 6.14	14.71 ± 6.14	NS	NS	NS
Jitter (%)	1.05 ± 3.07	0.38 ± 0.88	*∗*	NS	NS
*F*0_high_ (Hz)	605.84 ± 280.66	641.09 ± 316.71	*∗∗∗*	*∗∗∗*	*∗∗∗*
*F*0_low_ (Hz)	122.55 ± 35.25	124.32 ± 37.24	NS	*∗∗∗*	*∗∗∗*
*F*0_max_ (Hz)	482.66 ± 270.45	516.24 ± 305.99	*∗∗*	*∗∗∗*	*∗∗∗*
*I*_low_ (dB)	50.42 ± 5.48	53.3 ± 5.56	*∗∗∗*	NS	NS
*I*_high_ (dB)	92.8 ± 11.13	93.46 ± 10.27	*∗*	NS	NS
*I*_max_ (dB)	42.37 ± 14.38	39.68 ± 13.64	*∗∗∗*	NS	NS
*I*_mean_ (dB)	20.81 ± 8.9	17.87 ± 7.7	*∗∗∗*	NS	NS
*P*_VRP_	175.4 ± 66.89	131.2 ± 43.59	*∗∗∗*	NS	NS
*A*_VRP_ (ST × dB)	627.91 ± 384.21	556.48 ± 344.1	*∗∗∗*	NS	NS

*A*_VRP_: area of the VRP; DSI: dysphonia severity index; *F*0_high_: the highest tone; *F*0_low_: the lowest tone; *F*0_max_: frequency range, *I*_low_: the lowest intensity; *I*_high_: the highest intensity; *I*_max_: dynamic range; *I*_mean_: mean dynamic range per semitone; MPT: maximum phonation time; NS: not significant; *P*_VRP_: perimeter of VRP; ST: semitones; VEM: vocal extent measure; VRP: voice range profile; ^*∗*^significant at *p* < 0.05; ^*∗∗*^significant at *p* < 0.01; and ^*∗∗∗*^significant at *p* < 0.001.
